# Oriented Growth of Neural Stem Cell–Derived Neurons Regulated by Magnetic Nanochains

**DOI:** 10.3389/fbioe.2022.895107

**Published:** 2022-05-23

**Authors:** Lin Xia, Chen Zhang, Kaiming Su, Jiangang Fan, Yuguang Niu, Yafeng Yu, Renjie Chai

**Affiliations:** ^1^ State Key Laboratory of Bioelectronics, Department of Otolaryngology Head and Neck Surgery, Zhongda Hospital, School of Life Sciences and Technology, Advanced Institute for Life and Health, Jiangsu Province High-Tech Key Laboratory for Bio-Medical Research, Southeast University, Nanjing, China; ^2^ Beijing Key Laboratory of Neural Regeneration and Repair, Capital Medical University, Beijing, China; ^3^ Department of Otolaryngology-Head and Neck Surgery, Shanghai Jiao Tong University Affiliated Sixth People’s Hospital, Shanghai, China; ^4^ Department of Otolaryngology Head and Neck Surgery, Sichuan Academy of Medical Science, Sichuan Provincial People’s Hospital, Chengdu, China; ^5^ Department of Ambulatory Medicine, The First Medical Center of PLA General Hospital, Beijing, China; ^6^ Department of Otolaryngology, First Affiliated Hospital of Soochow University, Suzhou, China; ^7^ Department of Otolaryngology Head and Neck Surgery, Sichuan Provincial People’s Hospital, University of Electronic Science and Technology of China, Chengdu, China; ^8^ Co-Innovation Center of Neuroregeneration, Nantong University, Nantong, China; ^9^ Institute for Stem Cell and Regeneration, Chinese Academy of Science, Beijing, China

**Keywords:** neural stem cells, magnetic nanochains, oriented growth, newly derived neurons, nerve regeneration

## Abstract

Neural stem cell therapy has become a promising cure in the treatment of neurodegenerative disorders. Owing to the anisotropy of the nervous system, the newly derived neurons need not only the functional integrity but also the oriented growth to contact with the partner cells to establish functional connections. So the oriented growth of the newly derived neurons is a key factor in neural stem cell–based nerve regeneration. Nowadays, various biomaterials have been applied to assist in the oriented growth of neural stem cell–derived neurons. However, among these biomaterials, the magnetic materials applied in guiding the neuronal growth are still fewer than the other materials, such as the fibers. So in this work, we developed the magnetic nanochains to guide the oriented growth of neural stem cell–derived neurons. With the guidance of the magnetic nanochains, the seeded neural stem cells exhibited a good arrangement, and the neural stem cell–derived neurons showed well-oriented growth with the orientation of the nanochains. We anticipated that the magnetic nanochains would have huge potential in stem cell–based nerve regeneration.

## Introduction

Neurodegenerative disorders are a series of diseases caused by degeneration or loss of neurons in the nervous system, such as the brain (Shariati et al., 2020). Acute neurodegenerations are always caused by the lesioned loss of neurons, triggered by the temporally separate brain hemorrhage. In comparison, chronic neurodegenerations always show a long period of progressive loss of specific or multiple neuronal cells in the brain, brainstem, and spinal cord, such as the Alzheimer’s disease (AD), amyotrophic lateral sclerosis (ALS), Parkinson’s disease, spinal muscular atrophy (SMA), and Huntington’s disease (HD) (Allan and Rothwell, 2001; Shi et al., 2017; Raza et al., 2019). Although these neurodegenerative disorders indicate the same neuronal pathologies, the loss mechanism of neuronal cells is multifaceted, thus hampering the designing of a therapeutic approach. Currently, the therapy based on stem cells has attracted cumulative attention toward the cure for neurodegenerative disorders. Stem cells could be classified as various cell types and possess excellent proliferation and differentiation potentials to generate specific cell lineages. This unique property exhibited great importance for regenerative medicine and tissue engineering.

Along with the wide application of stem cell therapy, many biomaterials have been developed to assist the regenerative application of stem cells, such as the scaffolds. He et al. developed a kind of immunoregulatory hydrogel scaffold, which could be applied to promote the spinal cord injury. This immunoregulatory hydrogel scaffold could effectively scavenge the damage-associated molecular patterns and release the anti-inflammatory cytokine 10 (Shen et al., 2022). Qi et al. (2022) reported a dual-drug (cetuximab and FTY720) enhanced hydrogel, which could be introduced by injection. This kind of hydrogel could be integrated with neural stem cells and promote tissue regeneration in a complete transected rat SCI model. Luo et al. (2022) integrated chondroitin sulfate, gelatin, and polypyrrole to fabricate the injective hydrogel with excellent conductivity. After fabrication, the composite hydrogel was applied to promote the traumatic SCI repair. It was found that the composite hydrogel activated the neurogenesis of the endogenous neural stem cells, and the myelinated axon regeneration was also induced through the activation of PI3K/AKT and MEK/ERK pathways. So it could be concluded that biomaterials have been widely applied in nerve regeneration based on stem cell therapy.

**FIGURE 1:**
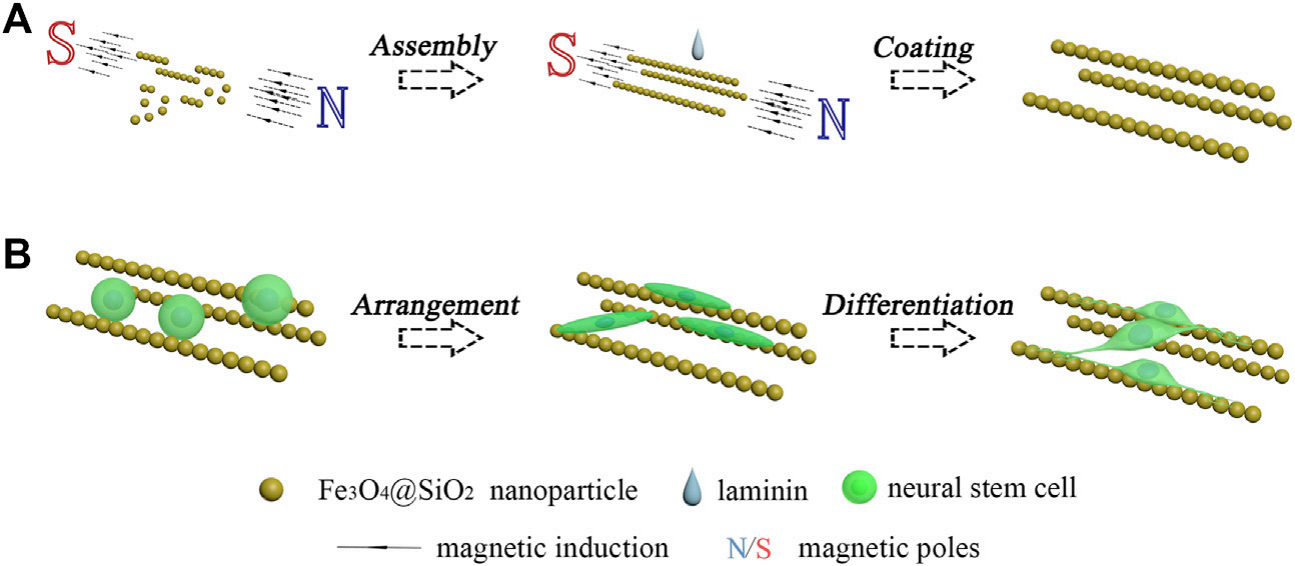
Schematic illustration of the oriented growth of newly derived neurons. **(A)** Assembly of magnetic nanochains. **(B)** Guided oriented growth of the newborn neurons derived from the seeded neural stem cells.

Among the various biomaterials that are applied in nerve regeneration, magnetic nanoparticles have attracted much attention. The magnetic nanoparticles possess a large specific surface area, good surficial modification, and excellent supermagnetism. These properties endowed a wide range of applications of the magnetic nanoparticles in biomedicine, such as the use of Fe_3_O_4_ nanoparticles in magnetic resonance imaging (Gradinaru et al., 2021; Wang et al., 2021; Khani et al., 2022). The good magnetic response makes the magnetic nanoparticles a good assembling property under the magnetic field (Yu et al., 2022). Many kinds of magnetic assemblies have been developed and applied in various fields, including nanochains (Luo et al., 2020; Cai et al., 2022; Qiao et al., 2022), nanosheets (Qin et al., 2021; Huang et al., 2022), and nanocrystals (Pan et al., 2021; Pileni, 2021; Deng et al., 2022). However, the application of magnetic assemblies in nerve regeneration has not been sufficiently studied. In this work, the Fe_3_O_4_ nanoparticles were coated with a thin layer of silicon dioxide. Then, the dispersed Fe_3_O_4_@SiO_2_ nanoparticles were arranged by the magnetic field to form the magnetic nanochains and immobilized by the coating of laminin. The neural stem cell monodispersed solution was dripped onto the immobilized magnetic nanochains. After adhesion, proliferation and differentiation culturings were sequentially performed. As shown in Figure 1, it was found that the adhered neural stem cells exhibited well-oriented arrangement, and the newly derived neurons grew along the orientation of the magnetic nanochains.

## Results

### Magnetic Material Characterization

**FIGURE 2:**
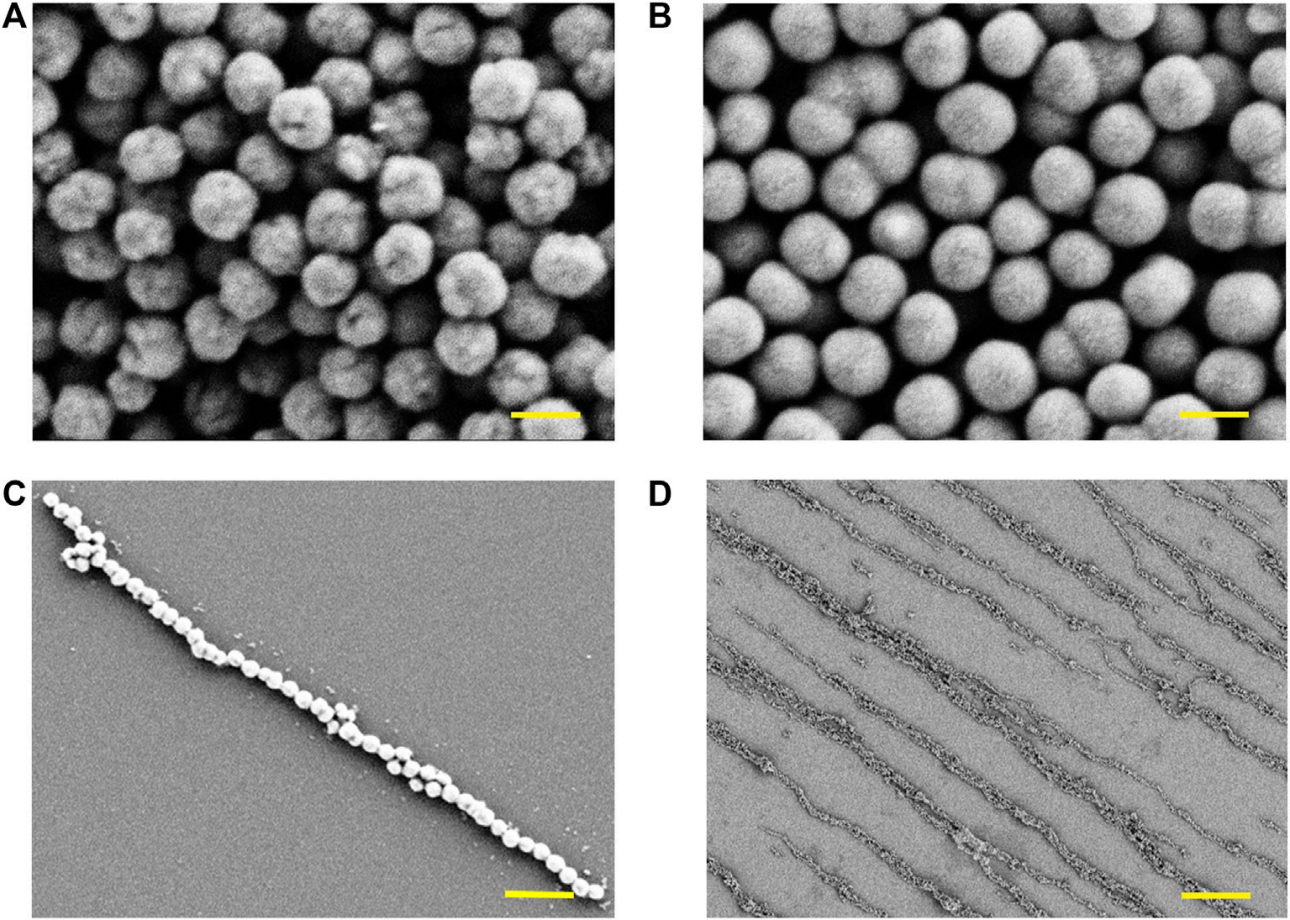
Characterization of magnetic nanomaterials. **(A)** Fe_3_O_4_ cores of the magnetic nanoparticles observed by SEM, and the scale bar is 200 nm. **(B)** Fe_3_O_4_@SiO_2_ nanoparticles observed by SEM, and the scale bar is 200 nm. **(C)** Representative single magnetic nanochain observed by SEM, and the scale bar is 1 μm. **(D)** Overview of magnetic nanochains observed by SEM, and the scale bar is 10 μm.

In this work, the magnetic nanochains were fabricated by the assembly of the dispersed solution of magnetic nanoparticles induced by the static magnetic field. The magnetic nanoparticles were obtained through hydrothermal synthesis, following with the surficial modification of SiO_2_. As shown in Figure 2, the Fe_3_O_4_ cores of the nanoparticles exhibited obvious spherical morphology (Figure 2A), and their surfaces presented obvious wrinkles. Then, the Fe_3_O_4_ nanoparticles were packaged by a SiO_2_ layer through surficial modification (Figure 2B). The surfaces of the Fe_3_O_4_@SiO_2_ nanoparticles were smooth in comparison with the Fe_3_O_4_ nanoparticles. After the manufacture of the magnetic nanoparticles, the magnetic nanochains were assembled by inducing of the magnetic field. The magnetic nanoparticles were arranged according to the magnetic induction lines and exhibited good linear topography. A representative single magnetic nanochain was presented in Figure 2C, and the magnetic nanochain exhibited good anisotropic morphology. Then, the laminin was applied to coat the assembled magnetic nanochains. The laminin is a kind of adhesion protein derived from the basement membrane, which is applied to promote adhesion, migration, and differentiation of cells. With coating, the magnetic nanochains were immobilized, maintaining the anisotropic morphology in the absence of the magnetic field. Moreover, the coating of laminin also improved the biocompatibility of the magnetic nanochains. The overview in Figure 2D presented the aligned magnetic nanochains, which possessed good anisotropic topography.

### Neural Stem Cells’ Oriented Arrangement

**FIGURE 3:**
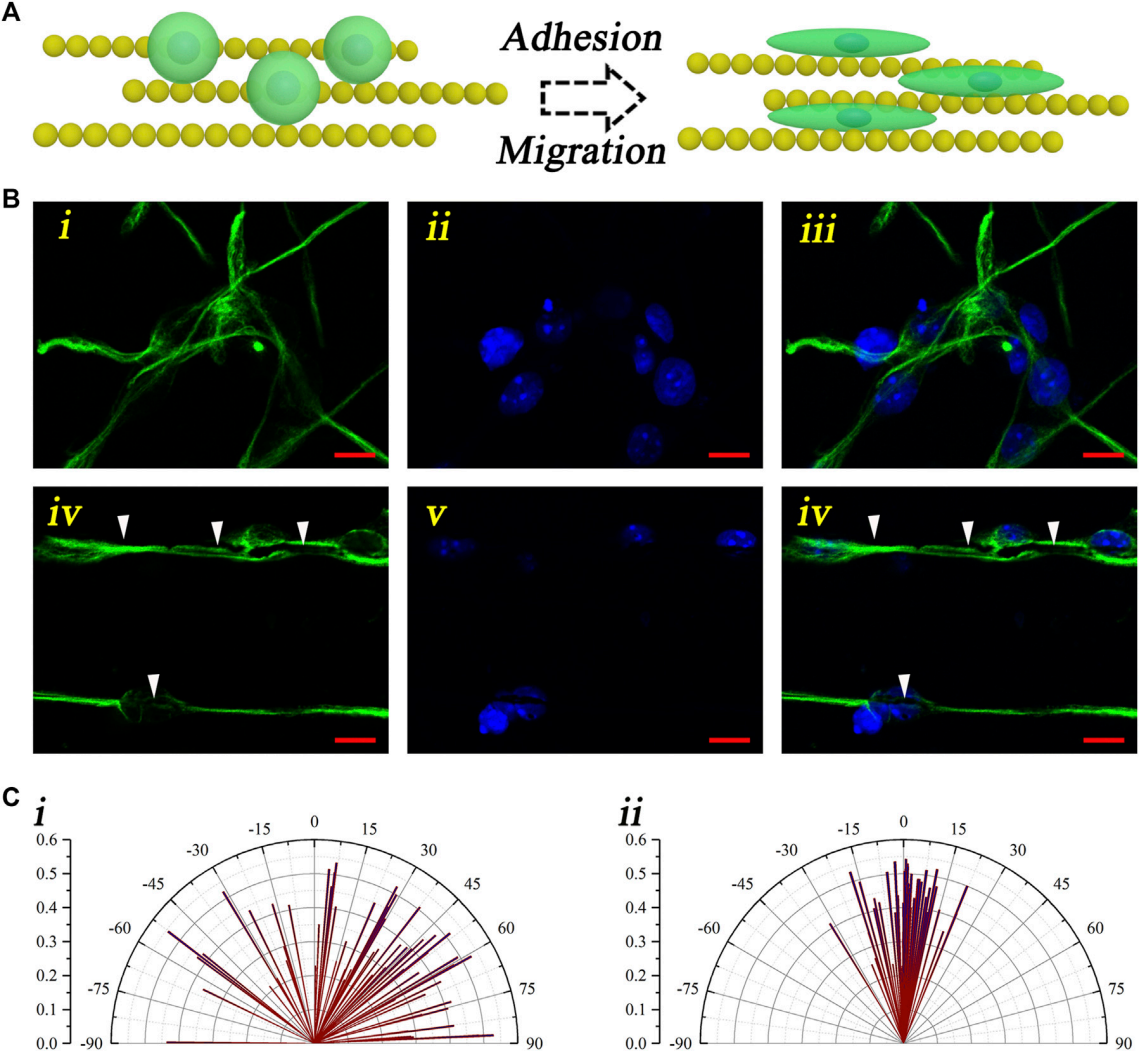
Oriented arrangement of the neural stem cells. **(A)** Schematic illustration of the neural stem cell–oriented arrangement under the guidance of magnetic nanochains. **(B)** Representative fluorescent photographs of neural stem cells both in the control (i–iii) and the nanochain-guided groups (iv–vi) (green: nestin; blue: DAPI), and the scale bars are 10 μm. **(C)** Orientation angle statistic graphs of both the control (i) and the nanochain-guided groups (ii).

To evaluate the guiding effect of the magnetic nanochains, the neural stem cell monodispersed solution was dripped onto the control coverslips and the magnetic nanochain–adhered coverslips. After culturing for 7 days, the neural stem cell activity marker “nestin” was applied to stain the cultured cells, thus visualizing the neural stem cells and evaluating the activity. As shown in Figure 3, the schematic illustration described the guidance of the neural stem cells performed by the magnetic nanochains (Figure 3A). With the guidance of the magnetic nanochains, the seeded neural stem cells exhibited well-oriented arrangement, according to the orientation of the magnetic nanochains. It could be found that the control neural stem cells were random after 7 days of culturing; in comparison, the nanochain-guided neural stem cells showed well-oriented arrangement to the orientation of the magnetic nanochains after 7 days of culturing (Figure 3B). The white arrows in Figure 3Biv–vi mark the magnetic nanochains. In the photograph, the magnetic nanochains interacted with the neural stem cells, and their orientations were highly consistent with each other. These results demonstrated a good “contact-guidance” effect of the magnetic nanochains in guiding the neural stem cell–oriented arrangement. In statistics, the nanochain-guided neural stem cells performed more than 70% orientation angles less than 10°. In comparison, the orientation angles of the control group were distributed randomly; there were only 12.7% of neural stem cells with the orientation angles less than 10° (Figure 3C **)**. Thus, it could be concluded that the magnetic nanochains with good anisotropic topography showed excellent oriented guidance on the neural stem cell arrangement by the “contact-guidance” effect, while the immunofluorescent results of the activity marker “nestin” also demonstrated that the neural stem cells maintained a good activity at the same time.

### Newly Derived Neurons’ Oriented Growth

**FIGURE 4:**
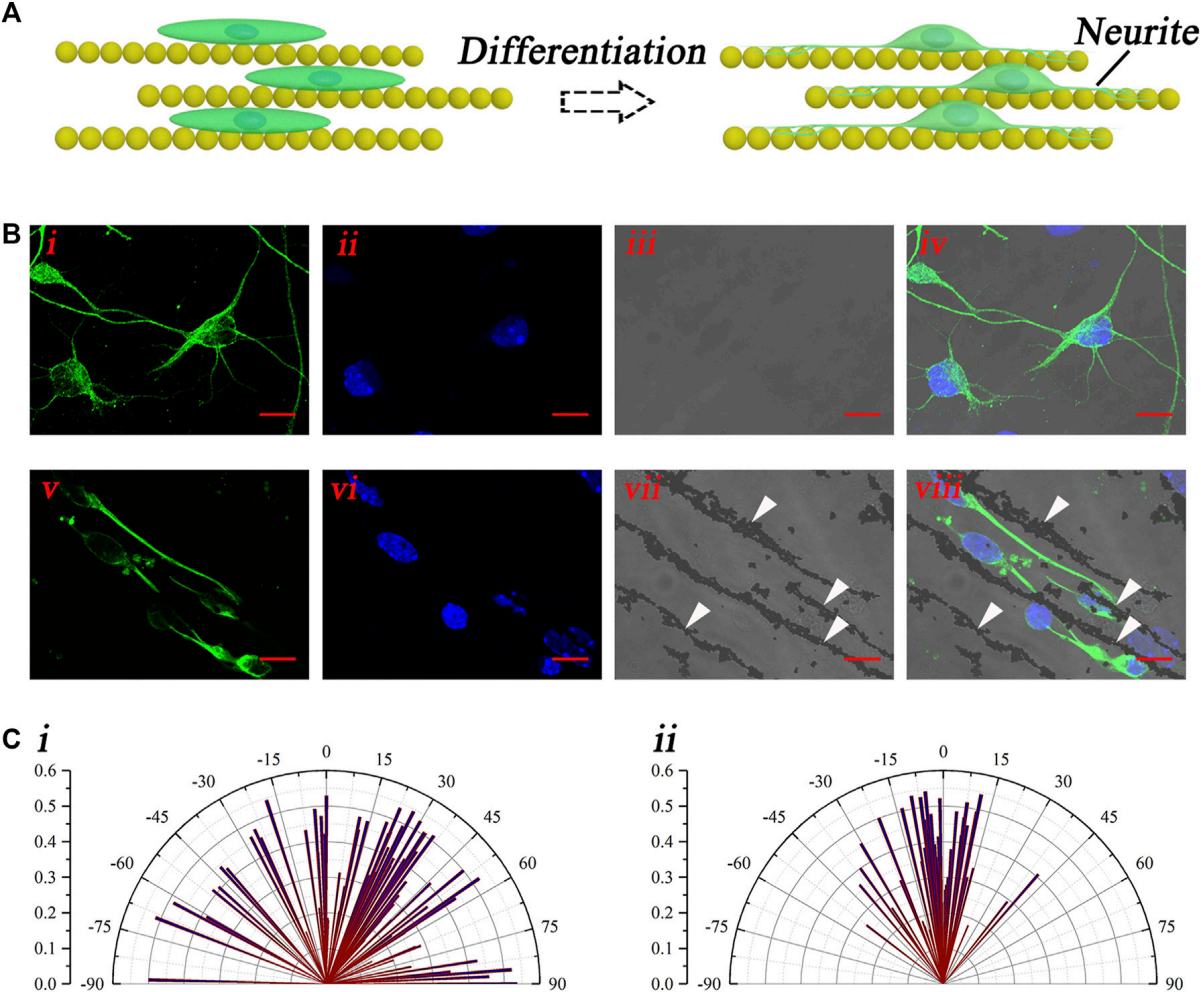
Oriented growth of the newly derived neurons. **(A)** Schematic illustration of the oriented growth of newly derived neurons. **(B)** Representative fluorescent photographs of newly derived neurons of both the control (i–iv) and the nanochain-guided (v–viii) groups, the scale bars are 10 μm. **(C)** Orientation angle statistic graphs of both the control (i) and the nanochain-guided (ii) newly derived neurons.

The aforementioned results have demonstrated the good guidance of the magnetic nanochains on the neural stem cell–oriented arrangement. After the arrangement, the differentiation culturing was performed by the replacement of the differentiation medium. Then, the orientation of the growth of the neural stem cell–derived neurons was evaluated. The schematic illustration in Figure 4A shows the oriented growth of the neural stem cell–derived neurons, with the guidance of the magnetic nanochains. With differentiation, the neurites of newly derived neurons outgrew and extended along with the magnetic nanochains, which exhibited well-oriented growth. To visualize the newly derived neurons, the marker of neuron TuJ-1 was applied to stain the differentiated cells, as shown in Figure 4B. The fluorescent photographs in Figure 4Bi–iv showed the control newly derived neurons, whose orientations were random. In comparison, the fluorescent photographs in Figure 4Bv–viii showed the nanochain-guided newly derived neurons. It could be found that the neurites of the newly derived neurons extended well, along with the magnetic nanochains. The white arrows marked the magnetic nanochains. Furthermore, to evaluate the guiding effect of the magnetic nanochains on the newly derived neurons, the orientation angles of the newly derived neurons were calculated both in the control and the nanochain-guided groups. As shown in Figure 4C, the statistic graphs showed that the orientation angles of the control newly derived neurons were random; in comparison, the orientation angles of the nanochain-guided newly derived neurons were highly concentrated in distribution. There were more than 50% of newly derived neurons with the orientation angle within 10° to the magnetic nanochains in comparison with 16.8% of the control newly derived neurons. These results demonstrated the good guiding effect of the magnetic nanochains on the oriented growth of the neural stem cell–derived neurons.

## Discussion

In this work, we have developed a guiding approach for the oriented growth of newly derived neurons based on magnetic nanochains. During fabrication, the magnetic nanoparticles were assembled into linear nanochains by inducing of the magnetic field. Based on the “contact-guidance” of the magnetic nanochains, the seeded neural stem cells exhibited a linear arrangement in comparison to the control. When aligned, neural stem cells maintained the good activity at the same time. This result demonstrated that the anisotropic topography of the magnetic nanochains possessed an excellent guiding effect in regulating the neural stem cells migration through the “contact-guidance” effect. Following the arrangement, the differentiation culturing of the neural stem cells was performed. With culturing, the neurites of the newly derived neurons extended along with the magnetic nanochains. By immunofluorescence staining, the newly derived neurons showed an obvious orientation in comparison to the control, as confirmed by the statistics. So it could be concluded that the magnetic nanochains also had a good guiding effect on the extension of the neurites of the newly derived neurons. This result exhibited huge potential in various anisotropic regenerative environments.

In addition, the magnetic nanochains were assembled by the Fe_3_O_4_@SiO_2_ magnetic nanoparticles, whose bioeffects and biosafety have been extensively studied and widely applied in biomedicine, such as magnetic resonance imaging (MRI). This property endowed excellent biocompatibility for the magnetic nanochains, which was confirmed by activity marker staining. Moreover, the Fe_3_O_4_@SiO_2_ magnetic nanoparticles possessed good monodispersity, which was very convenient to prepare the monodispersed solution. This monodispersed solution could easily be introduced to the regenerative site, which possessed huge potential in clinical application. Furthermore, the magnetic nanochains could be easily integrated with the other biomaterials, such as the hydrogel, to fabricate the composite materials. During fabrication, the linear topography of the magnetic nanochains could be effectively integrated into the composite biomaterials, thus significantly enhancing the guiding effect of the composite biomaterials. So it could be concluded that the magnetic nanochains would become a versatile platform in biomedical applications.

## Materials and Methods

### Materials

Ethylene glycol, ferric chloride, and sodium acetate were obtained from Aladdin (Shanghai, China). Sodium hydroxide and tetraethoxysilane (TEOS) were obtained from Macklin (Shanghai, China). Laminin and acrylamide were obtained from Sigma-Aldrich (MO, United States). Phosphate buffered saline (PBS), accutase, Hanks’ balanced salt solution (HBSS), B-27, and recombinant human epidermal growth factor (EGF) were obtained from Gibco (NY, United States). Recombinant murine FGF-basic (FGF) was obtained from PropTech (NJ, United States). The NeuroCult™ differentiation kit was obtained from STEMCELL Technologies (CA, United States). The nestin marker (Nestin antibody, AN205), TuJ-1 marker (neuronal class III β-tubulin, AT809), and DAPI (C1002) were obtained from Beyotime (Jiangsu, China). Donkey anti-mouse secondary antibody (A21202) was obtained from ThermoFisher (MA, United States).

### Fabrication of the Fe_3_O_4_ Nanoparticles

In detail, 16 ml of ethylene glycol, 0.26 g of ferric chloride, 1.2 g of sodium acetate, 0.4 g of poly (4-styrenesulfonic acid-co-maleic acid) sodium salt, 4.5 mg of acrylamide, and 20–50 μL of deionized water were added into a boiling flask in sequence. Then, the mixed solution was kept for vigorous stirring for 30 min to obtain a solution with uniform yellow under room temperature. Then, 0.24 g sodium hydroxide was added to the uniform yellow solution, maintaining the stirring until the mixed solution became black. The black solution was transferred into a 20-ml stainless steel autoclave lined by Teflon and kept under 190°C for 9 h. After the process, the solution was cooled to room temperature, and the obtained Fe_3_O_4_ nanoparticles were collected with a magnet. Then, the Fe_3_O_4_ nanoparticles were sequentially washed by the mixed solution of deionized water and ethanol (1:1) three times and then by deionized water three times. Then, the Fe_3_O_4_ nanoparticles were dispersed in deionized water in the final step. The manufacturing procedure was according to the hydrothermal synthesis, as elaborated in the literature (Dong et al., 2016; Tang et al., 2018; Zhang et al., 2019).

### Fabrication of the Fe_3_O_4_@SiO_2_ Nanoparticles

First, 6 ml of Fe_3_O_4_ dispersed solution (obtained above) was dissolved in 40 ml ethanol, and then, 2 ml of ammonia hydroxide was added to the solution. It was followed by sonicating the mixed solution for 5 min and transferring the mixed solution into a three-necked flask. It was subjected to stirring for 10 min with the speed of 600 rpm under a 50°C water bath. Then, TEOS was added into the mixed solution at a speed of 200 μL per 20 min. Under constant stirring for 1 h, the manufactured Fe_3_O_4_@SiO_2_ nanoparticles were collected by a magnet, washed with ethanol and deionized water, and then dispersed into deionized water.

### Magnetic Nanochain Fabrication

The monodispersed solution of Fe_3_O_4_@SiO_2_ nanoparticles (20 μg/ml) containing 1% laminin was added onto a coverslip within a 20 mT static magnetic field. The whole device was maintained at 37°C with 5% CO_2_ overnight. The Fe_3_O_4_@SiO_2_ nanoparticles were arranged in lines, according to the magnetic induction. Then, laminin coating was performed on the aligned magnetic nanochains. After coating, the magnetic nanochains were immobilized without the magnetic field.

### Isolation and Proliferation of Neural Stem Cells

The hippocampuses of embryonic rats (16–19 days) were collected into HBSS solution in a Petri dish under 4°C. Then, it was followed by discarding the HBSS solution and dripping the accutase into the collected hippocampuses for digestion. Digestion was allowed for 20 min at 37°C with 5% CO_2_. The accutase was discarded. Then, the proliferation medium (DMEM/F12 containing 2% B-27, 20 ng/ml FGF, and 20 ng/ml EGF) was added to the Petri dish, and the hippocampuses were triturated gently to form the monodispersed cell solution. Then, the monodispersed cell solution was cultured in the incubator at 37°C with 5% CO_2_.

### Differentiation of Neural Stem Cells

The neural stem cell monodispersed solution was dripped onto the laminin-coated magnetic nanochains and cultured for 7 days with the proliferation medium. Then, the medium was displaced by the differentiation medium (NeuroCult™ differentiation kit). The differentiation culturing was maintained for 7 days. The neural lineage cells were stained by immunofluorescence and observed by using a confocal microscope.

## Data Availability

The raw data supporting the conclusions of this article will be made available by the authors, without undue reservation.
